# Hyperglycemia and steroid use increase the risk of rhino-orbito-cerebral mucormycosis regardless of COVID-19 hospitalization: Case-control study, India

**DOI:** 10.1371/journal.pone.0272042

**Published:** 2022-08-08

**Authors:** Manickam Ponnaiah, Sivaraman Ganesan, Tarun Bhatnagar, Mahalakshmy Thulasingam, Marie Gilbert Majella, Mathan Karuppiah, S. A. Rizwan, Arun Alexander, Sonali Sarkar, Sitanshu Sekhar Kar, Tamilarasu Kadhiravan, Aparna Bhatnagar, Prasanna Kumar S., Vivekanandan M. Pillai, Pradeep Pankajakshan Nair, Rahul Dhodapkar, Pampa Ch Toi, Rakesh Singh, Nirupama Kasthuri, Girish C. P. Kumar, Saranya Jaisankar, Vaibhav Saini, Ankita Kankaria, Anuradha Raj, Amit Goyal, Vidhu Sharma, Satyendra Khichar, Kapil Soni, Mahendra Kumar Garg, Kalaiselvi Selvaraj, ShriKrishna B. H., Kranti Bhavana, Bhartendu Bharti, C. M. Singh, Neha Chaudhary, Vijayaravindh R., Gopinath K., Karthikeyan Palaninathan, Simmi Dube, Rita Singh Saxena, Nikhil Gupta, A. Rathinavel, S. Priya, Arulsundareshkumar, Shama A. Bellad, Avinash Kavi, Anilkumar S. Harugop, Kailesh Pujary, Kirthinath Ballala, Sneha Deepak Mallya, Hanumanth M. Prasad, D. Ravi, N. K. Balaji, Raghuraj Hegde, Neha Mishra, Shalina Ray, S. Karthikeyan, Sudha Ramalingam, A. Murali, Sudhakar Vaidya, Mohit Samadhiya, Dhaval Bhojani, Somu Lakshmanan, Sudagar R. B. Singh, Nataraj Pillai, P. Deepthi, K. Banumathi, V. Sumathi, D. Ramesh, Sonam Poonam Nissar, Khushnood M. Sheikh, Manisha N. Patel, Vipul Shristava, Suresh S. Kumar, K. Shantaraman, Rajkamal D. Pandian, Manoj Murhekar, Rakesh Aggarwal

**Affiliations:** 1 ICMR-National Institute of Epidemiology, Chennai, India; 2 Jawaharlal Institute of Postgraduate Medical Education and Research, Puducherry, India; 3 Apollo Specialty Hospitals, Vanagaram & Billroth Hospital, Shenoy Nagar, Chennai, India; 4 Sri Ramachandra Medical Centre, Chennai, India; 5 All India Institute of Medical Sciences, Bathinda, India; 6 All India Institute of Medical Sciences, Jodhpur, India; 7 All India Institute of Medical Sciences, Nagpur, India; 8 All India Institute of Medical Sciences, Patna, India; 9 G Kuppuswamy Naidu Memorial Hospital, Coimbatore, India; 10 Gandhi Medical College, Bhopal, India; 11 Government Rajaji Hospital and Medical College, Madurai, India; 12 Jawaharlal Nehru Medical College, Belagavi, India; 13 Kasturba Medical College, Manipal, India; 14 Mandya Institute of Medical Sciences, Mandya, Karnataka, India; 15 Manipal Hospitals, Bangalore, India; 16 PSG Institute of Medical Sciences and Research, Coimbatore, India; 17 Ruxmaniben Deepchand Gardi Medical College, Ujjain, India; 18 SRM Institutes for Medical Science, Chennai, India; 19 Sundaram Medical Foundation, Chennai, India; 20 Surat Municipal Institute of Medical Education and Research, Surat, India; 21 Tirunelveli Medical College, Tirunelveli, India; SRM Institute of Science and Technology, INDIA

## Abstract

**Background:**

In the ongoing COVID-19 pandemic, an increased incidence of ROCM was noted in India among those infected with COVID. We determined risk factors for rhino-orbito-cerebral mucormycosis (ROCM) post Coronavirus disease 2019 (COVID-19) among those never and ever hospitalized for COVID-19 separately through a multicentric, hospital-based, unmatched case-control study across India.

**Methods:**

We defined cases and controls as those with and without post-COVID ROCM, respectively. We compared their socio-demographics, co-morbidities, steroid use, glycaemic status, and practices. We calculated crude and adjusted odds ratio (AOR) with 95% confidence intervals (CI) through logistic regression. The covariates with a p-value for crude OR of less than 0·20 were considered for the regression model.

**Results:**

Among hospitalised, we recruited 267 cases and 256 controls and 116 cases and 231 controls among never hospitalised. Risk factors (AOR; 95% CI) for post-COVID ROCM among the hospitalised were age 45–59 years (2·1; 1·4 to 3·1), having diabetes mellitus (4·9; 3·4 to 7·1), elevated plasma glucose (6·4; 2·4 to 17·2), steroid use (3·2; 2 to 5·2) and frequent nasal washing (4·8; 1·4 to 17). Among those never hospitalised, age ≥ 60 years (6·6; 3·3 to 13·3), having diabetes mellitus (6·7; 3·8 to 11·6), elevated plasma glucose (13·7; 2·2 to 84), steroid use (9·8; 5·8 to 16·6), and cloth facemask use (2·6; 1·5 to 4·5) were associated with increased risk of post-COVID ROCM.

**Conclusions:**

Hyperglycemia, irrespective of having diabetes mellitus and steroid use, was associated with an increased risk of ROCM independent of COVID-19 hospitalisation. Rational steroid usage and glucose monitoring may reduce the risk of post-COVID.

## Introduction

Mucormycosis is a rare opportunistic angio-invasive fungal infection associated with high morbidity and mortality. Several clinical presentations of mucormycosis have been described depending on the site of involvement, the most common being rhino-orbito-cerebral mucormycosis (ROCM). Globally, reported incidence (per million population) ranges from 0·005 to 1·7 in the pre-Coronavirus disease 2019 (COVID-19) era, and in India, the incidence is 140, about 80 times higher than reported in developed countries [1]. This higher burden could be due to the relatively significant burden of uncontrolled diabetes mellitus [2].

Rapid progression of Mucormycosis makes its early diagnosis and treatment critical, considering that a delay of even six days doubles 30-day fatality from 32% to 66% [3]. Tissue necrosis resulting from angioinvasion and vascular thrombosis is a hallmark of the disease but is a late sign. Patients with diabetes, on steroids, with immunocompromised states such as haematological malignancy, hematopoietic/solid organ cell transplantation, treatment with deferoxamine, acquired immunodeficiencies, trauma, and malnutrition are at high risk for mucormycosis [4, 5]. A high index of suspicion is necessary for them, especially if they report unilateral facial pain or swelling, orbital swelling or proptosis.

In the context of the ongoing COVID-19 pandemic in India, reports highlighted the increased incidence of ROCM, otherwise a rare entity [6–8]. The risk of COVID-19 associated ROCM is reported to be higher among men, diabetics, more so with poor glycemic control, steroid users, and severe COVID-19 [5, 6, 8–11]. The usual time of diagnosis with mucormycosis among COVID-19 varied from that of a concurrent infection to even seven weeks post COVID-19 diagnosis [9]. Towards the end of June 2021, the Indian health ministry reported over 40,000 mucormycosis cases and mandated its notification while several Indian states declared it an epidemic [10]. The upsurge of mucormycosis amidst the ongoing pandemic was hypothesized to be driven by the interplay between host factors like weakened immunity following COVID-19, poorly controlled diabetes mellitus, rampant use of steroids/immunomodulators, and other factors like prolonged hospital stay, continuous oxygen support, poor hospital infection control, and nursing care practices [11]. Indian researchers documented various factors linked with COVID-19 associated mucormycosis, including steroid use, diabetes mellitus, higher serum ferritin, and hypoxemia [12, 13]. Few studies have focused on highly immunocompromised groups [14] and individual-level risk factors such as zinc supplementation [15] and mean serum iron and total iron-binding capacity [16]. However, many of these studies lacked adequate sample size, did not comprehensively evaluate the range of risk factors, and did not specifically generate evidence on differential factors operating for PC-ROCM by their COVID-19 specific hospitalization status and factors thereof. Such studies may inform ways to be prepared and thus reduce the burden of PC-ROCM. Therefore, we conducted a multicentric study to determine the risk factors for PC-ROCM separately among those ever and never-hospitalised for COVID-19 management.

## Methods

### Study design and setting

We designed and conducted an unmatched case-control study in 20 tertiary care hospitals from the public and private sectors providing management for COVID-19 and ROCM across India during June and July 2021.

### Study participants

#### Test positive COVID-19

Those testing positive for Severe Acute Respiratory Syndrome (SARS)-Coronavirus (CoV)-2 through rRT-PCR/TrueNat/ GeneXpert/Rapid Antigen Test.

#### Post COVID rhino-orbito-cerebral mucormycosis (PC-ROCM)

Individuals diagnosed with ROCM clinically and/or based on diagnostic nasal endoscopy and/or contrast-enhanced MRI/CT scan with laboratory confirmation on direct microscopy (KOH/ Calcofluor white) or culture or histopathology or molecular diagnostic methods (diagnosed after April 1, 2021) and tested positive for COVID-19 any time between 12 weeks to 1 day before ROCM diagnosis.

#### Case for the ever-hospitalized group

Patients who had PC-ROCM and received hospitalised COVID-19 care, irrespective of the duration of hospitalization.

#### Controls for the ever-hospitalized group

Patients who had the negative history of ROCM symptoms and received hospitalised COVID-19 care, irrespective of the duration of hospitalization.

#### Case for the never-hospitalized group

Patients who had PC-ROCM and received COVID-19 care at home/COVID care centre only.

#### Control for the never-hospitalized group

Patients who had a negative history of ROCM symptoms and received COVID-19 care at home/COVID care centre only.

*Sample size for case-control study among ever-hospitalised for COVID-19*. We needed 256 cases and controls each based on assumptions that 68% of the hospitalised COVID-19 patients were treated with steroids [17], odds ratio (OR) of at least two for steroid use, alpha error of 5%, power of 80%, 1:1 ratio of cases to controls and 25% non-response.

*Sample size for case-control study among never-hospitalised for COVID-19*. We needed 108 cases and 216 controls for the assumptions that 15% of the never-hospitalised COVID-19 patients were treated with steroids, expected OR of at least three for steroid use, alpha error of 5%, power of 80%, the ratio of cases: controls to be 1:2 and 25% non-response.

We used OpenEpi Online calculator version 3·01 for sample size calculation (https://www.openepi.com/SampleSize/SSCC.htm).

### Selection of cases and controls

Cases were recruited from those who received care for ROCM from the study hospital. Controls were recruited from those tested positive for COVID-19 at the study hospital and on the same date as the case. We excluded those were 18 years of age, without information on COVID-19 hospitalisations/management, and who were not traceable. Each study hospital created a sampling frame of cases and controls separately for hospitalised and never-hospitalised groups (during COVID-19 illness). We selected controls through simple random sampling from the list of patients who tested positive for COVID-19 on the same date as cases through a custom-built web tool [18].

### Data collection and data quality management

We developed case record forms (CRFs) to gather data on exposure (a) from the time of diagnosis of COVID-19 until discharge from the hospital/home care and (b) from the time of discharge from the hospital for COVID/19 until the development of PC-ROCM. Trained site investigators interviewed the cases in the hospital and abstracted treatment and diagnostic information from hospital records. For controls, data was collected through telephone interviews, and relevant clinical information was abstracted from hospital records/prescriptions. The filled-in CRFs were shared online with the I-MUCOR study group for data entry into Research Electronic Data Capture (REDCap) software (https://www.project-redcap.org/).

### Statistical analysis

We used descriptive statistics (percentages), means (standard deviation), median (inter-quartile range) to describe cases and controls. We converted steroid dose into prednisolone equivalents [19] and used the median cumulative dose among the controls to categorize the variable into low and high steroid use. We used the median duration of hospitalisation among controls to dichotomize the cases. While evaluating the blood sugar on PC-ROCM, the glycaemic status during the COVID-19 illness is crucial, rather than HbA1C, which would depict the past control, including the period before COVID-19 infection. Hence, we defined hyperglycemia as random blood sugar value above 200 mg/dl and fasting blood sugar above 126mg/dl, at least once during COVID-19 management was considered as elevated plasma glucose.

We used logistic regression to calculate crude and adjusted odds ratios (AOR) with 95% CI. For each of the ever- and never-hospitalised groups we selected covariates with a p-value for crude OR of less 0·20 for consideration in the multiple regression model [20]. The variables rural/urban residence, type of house, and occupation were collinear. Hence occupation was chosen for further analysis.

**Fig 1:**
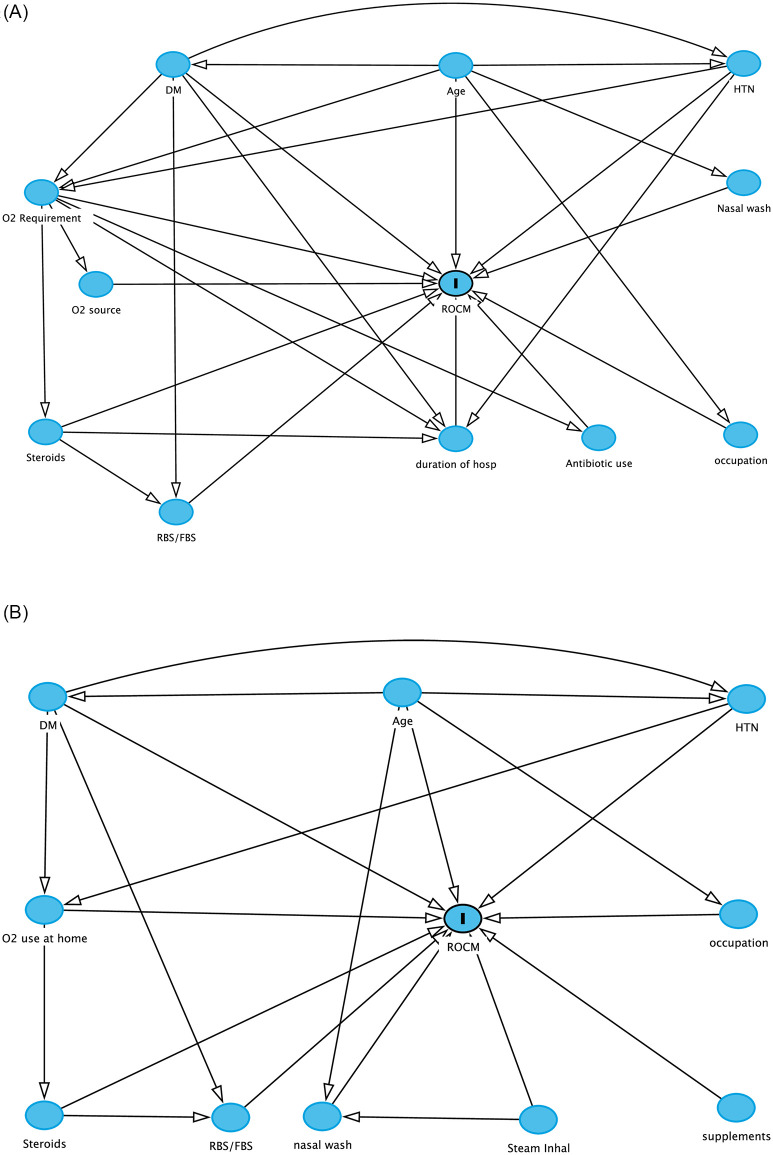
Directed acyclic graph of Post COVID-19 rhino orbito cerebral mucormycosis for (a) ever-hospitalised and (b) never-hospitalised patients.

Using these variables, we constructed directed acyclic graphs (DAG) separately for the never- and ever-hospitalized using DAGitty software (http://www.dagitty.net/) [21] to visually represent the causal relationships between various risk factors and PC-ROCM. (Fig 1) The causal relationships were assumed based on literature review, and contextual understanding arrived at by consensus among the research team, including specialists in medicine, otorhinolaryngology, epidemiology, and public health. We used the backdoor criterion to identify the minimal set of covariates as potential confounders that needed to be adjusted for each risk factor separately [21]. We compared the -2 log-likelihood ratios of models with and without potential confounders to arrive at the final list of covariates to be adjusted for each risk factor using multiple logistic regression. We did not observe any difference between the effect estimates obtained with or without adjustment for ’study site’ (data not shown). We hence did not include it in the final logistic regression model. We used Stata ver. 16 (StataCorp LLC, Texas, USA) for the analyses.

### Human participants protection

The Institutional Human Ethics Committee of the lead institutions of I-MUCOR and all the study hospitals approved the study procedures. The treating physicians of the study hospitals recruited study participants after explaining the study procedures and obtaining written informed consent directly from the cases and through digital modes from the controls.

## Results

### Among cases and controls ever-hospitalised for COVID-19

#### Profile of cases and controls (Table 1)

**Table 1:** Socio-demographic characteristics of the cases and controls, multi-centric case-control study of post-COVID rhino-orbito-cerebral mucormycosis, India, 2021.

Characteristics		Ever-hospitalised for COVID-19			Never-hospitalised for COVID-19		
		Cases (N = 267)	Controls (N = 256)	p value	Cases (N = 116)	Controls (N = 231)	p value
		n (%)	n (%)		n (%)	n (%)	
Age group* (in years)	18–44	75 (28·1)	108 (42·4)	0·002	37 (31·9)	169 (73·2)	<0·001
	45–59	129 (48·3)	90 (35·3)		53 (45·7)	44 (19·0)	
	≥ 60	63 (23·6)	57 (22·4)		26 (22·4)	18 (7·8)	
**Gender**	Female	89 (33·3)	97 (37·9)	0·276	41 (35·3)	98 (42·4)	0·204
	Male	178 (66·7)	159 (62·1)		75 (65·7)	133 (57·6)	
**Place of residence** ^†^	City or town	168 (63·2)	184 (71·9)	<0·001	66 (56·9)	181 (78·4)	<0·001
	Village	98 (36·8)	72 (28·1)		50 (43·1)	50 (21·6)	
**Occupation** ^‡^	Unemployed	103 (38·8)	96 (37·5)	0·101	42 (36·2)	79 (34·3)	0·026
	Agriculture	31 (11·6)	17 (6·7)		22 (19·0)	22 (9·6)	
	Other types of occupation	132 (49·6)	143(55·9)		52 (44·8)	129 (56·1)	
**Education** ^§^	No formal education	28 (10·0)	24 (9·0)	0·051	16 (13·8)	13 (5·6)	<0·001^#^
	Primary level	57 (21·0)	38 (15·0)		23 (19·8)	27 (11·7)	
	Secondary level	81 (30·0)	75 (29·0)		39 (33·6)	55 (23·9)	
	Graduate and above	101 (38·0)	118 (46·0)		38 (32·8)	135 (58·7)	
**Housing type** ^¶^	Pucca	249 (93·3)	248 (96·9)	0·057	98 (85·0)	222 (97·0)	<0·001
	Semi-pucca / kutcha	18 (6·7)	8 (3·1)		18 (15·0)	8 (3·0)	

Missing values:

*Age for 1 control in ever-hospitalised group;

^†^ Place of residence for 1 case in ever-hospitalised group;

^‡^ occupation for 1 case in ever-hospitalised and 1 control in never-hospitalised groups;

^§^ education for 1 control each in ever and never-hospitalised groups;

^¶^ Housing type for 1 control in never-hospitalised group, pucca house indicates solid and permanent structure, kutcha house indicates temporary structure.

We included 267 cases and 256 controls. More cases than controls (48% vs. 35%) were 45–59 years old. The majority of cases and controls were men (67% and 62%), resided in a city or town (63% vs. 72%), had completed secondary and graduate education (68% vs. 75%), and lived in pucca (solid and permanent structure) house (93% vs. 97%).

#### Analysis of risk factors for PC-ROCM (Table 2)

**Table 2:** Risk factors for mucormycosis among ever-hospitalised COVID-19 patients, multi-centric case-control study of post-COVID rhino-orbito-cerebral mucormycosis, India, 2021.

Characteristics		Cases (N = 267)	Controls (N = 256)	Odds Ratio (95% CI)			
				Crude estimate	p value	Adjusted estimate	p value
		n (%)	n (%)				
**SOCIO-DEMOGRAPHIC**							
Age group (years)*^†^	18–44	75 (28·1)	108 (42·4)	Reference		-	
	45–59	129 (48·3)	90 (35·3)	2·1 (1·4 to 3·1)	<0·001		
	≥ 60	63 (23·6)	57 (22·4)	1·6 (1·0 to 2·5)	0·050		
Occupation^‡^^$^	Agricultural	31 (11·6)	17 (6·6)	1·8 (1·0 to 3·4)	0·052	1·7 (0·9 to 3·2)	0·089
	Non-agricultural occupations & unemployed	235 (88·4)	239 (93·4)	Reference			
**CO-MORBIDITY**							
Presence of Diabetes Mellitus^†^		189 (70·8)	84 (32·8)	4·9 (3·4 to 7·1)	<0·001	-	
Presence of Hypertension^§^		86 (32·2)	60 (23·4)	1·6 (1·1 to 2·3)	0·026	1·0 (0·7 to 1·6)	0·923
Presence of any other comorbidities		24 (9·0)	24 (9·4)	0·9 (0·5 to 1·7)	0·873		
**CLINICAL STATUS/ MANAGEMENT**							
Elevated plasma glucose level^¶^^@^		162 (96·4)	80 (70·2)	11·5 (4·6 to 28·5)	<0·001	6·4 (2·4 to 17·2)	<0·001
Median Serum ferritin in μg/L (IQR)		585^††^ (297–796)	474^‡‡^ (166–723)		0·122		
**Steroid use** ^†^							
Ever used steroids		239 (89·5)	186 (72·7)	3·2 (2·0 to 5·2)	<0·001	-	
More than 480 mg prednisolone equivalent *{Median dose among controls}*		124(46·4)	89 (34·8)	3·5 (2·1 to 5·8)	<0·001	-	
Less than 480 mg prednisolone equivalent *{Median dose among controls}*		104(38·9)	93 (36·3)	2·8 (1·7 to 4·7)	<0·001	-	
Unknown dose		11 (4·1)	4 (1·6)	6·9 (2·0 to 23·4)	0·002	-	
No steroid use		28 (10·5)	70 (27·3)	Reference			
**Antibiotic use** ^†^		237 (88·8)	213 (83·2)	1·6 (1·0 to 2·6)	0·068	-	
More than 9 days of hospitalization^#^ *{Median among controls}*		141 (52·8)	120 (46·9)	1·3 (0·9 to 1·8)	0·175	0·8 (0·5 to 1·2)	0·241
**Received Oxygen support** **		177 (66·3)	156 (60·9)	1·3 (0·9 to 1·8)	0·203	1·0 (0·7 to 1·5)	0·859
**Source of Oxygen support among those who received oxygen support (n = 333)** ^†^							
Oxygen cylinder		57 (32·2)	19 (12·1)	3·5 (1·9 to 6·1)	<0·001	-	
Other sources of oxygen		120 (67·8)	137 (87·9)	Reference			
**Used other medications**							
Remdesivir		126 (47·2)	123 (48·1)	1·0 (0·7 to1·4)	0·845	-	
Ivermectin		103 (38·6)	95 (37·1)	1·1 (0·7 to1·5)	0·729	-	
Hydroxychloroquine		3 (1·1)	0	-	-	-	
Tocilizumab		2 (0·7)	2 (0·8)	1·0 (0·1 to 6·8)	1	-	
Iron supplements		12 (4·5)	7 (2·7)	1·7 (0·6 to 4·3)	0·29	-	
Zinc containing vitamin supplements*		204 (76·4)	211 (82·4)	0·7 (0·5 to 1·1)	0·090	-	
Indian traditional medicines (Ayush)		9 (3·4)	8 (3·1)	1·1 (0·4 to 2·8)	0·874	-	
**Nasal wash** ^‡^							
Did many times in a day		15 (5·6)	3 (1·2)	5·1 (1·5 to 17·9)	0·011	4·8 (1·4 to 17·0)	0·014
Once a day		17 (6·4)	12 (4·7)	1·5 (0·7 to 3·1)	0·336	1·4 (0·6 to 3·0)	0·399
Never done		235 (88·0)	241 (94·1)	Reference		Reference	
**Never practiced steam Inhalation**		186 (69·7)	189 (73·8)	1·2 (0·8 to 1·8)	0·291	-	
**Under home isolation prior to hospitalization**		119 (44·6)	106 (41·4)	1·1 (0·8 to 1·6)	0·465	-	
**Median (IQR) days of home isolation**		3 (2, 6)	4 (2, 6)		0·835		
**Oxygen use at home**		14 (5·2)	14 (5·5)	1·0 (0·4 to 2·0)	0·909	0·6 (0·3 to 1·5)	0·304
**MASK USE** ^†^							
**Prior to onset of COVID-19**							
Cloth masks only		105 (39·3)	98 (38·3)	0·7 (0·3 to 1·5)	0·374	-	
Surgical masks only		102 (38·2)	112 (43·7)	0·6 (0·3 to 1·3)	0·198	-	
Both Cloth and Surgical mask		43 (16·1)	35 (13·7)	0·8 (0·3 to 1·9)	0·609	-	
No masks		17 (6·4)	11 (4·3)	Reference		-	
**After hospitalisation**^&^							
Cloth masks only		59 (22·9)	67 (26·6)	0·6 (0·2 to 1·7)	0·356	-	
Surgical masks only		151 (58·5)	152 (60·3)	0·7 (0·3 to 1·9)	0·473	-	
Both Cloth and Surgical mask		38 (14·7)	26 (10·3)	1·0 (0·3 to 3·0)	0·967	-	
No masks		10 (3·9)	7 (2·8)	Reference			

* 1 missing data among cases;

^†^ No confounders identified;

^‡^ Adjusted for age;

^$^ 1 missing data among cases;

^§^ Adjusted for age and diabetes mellitus;

^¶^ Adjusted for diabetes mellitus, and Steroid use;

^@^ data missing for 99 cases and 142 controls;

^&^ data missing for 9 cases and 4 controls;

^#^ Adjusted for diabetes mellitus, hypertension, and steroid use;

** Adjusted for age, diabetes mellitus, and hypertension;

^††^ Missing values, n = 90;

^‡‡^ Missing values, n = 105;

^¥^ co-morbidities includes heart disease, chronic kidney disease, cancer, organ transplant, HIV/AIDS, chronic sinusitis

A significantly higher proportion of cases had a known history of diabetes mellitus (71% vs. 33%) than controls. Participants with diabetes mellitus were five times more likely to have PC-ROCM (AOR = 4·9; 95% CI = 3·4 to 7·1). A substantial number of non-diabetic cases and controls had elevated plasma glucose levels (97% and 53%, respectively, data not shown in the table). Hence, it necessitated the inclusion of plasma glucose as an independent variable in the multiple regression model. Elevated plasma glucose was six times more likely to be associated with PC-ROCM after adjusting for diabetes mellitus and steroid use (AOR = 6·4; 95% CI = 2·4 to 17·2; Fig 1). After adjusting for confounders, hypertension, occupation, duration of hospitalization, receipt of oxygen support, use of antibiotics, remdesivir, and ivermectin were not associated with PC-ROCM.

Steroid use, irrespective of the cumulative dose, was significantly associated with PC-ROCM (OR = 3·2; 95% CI: 2·0 to 5·2) (Table 2). No dose-response relationship was documented between the amount of steroid use and duration of hospitalization with PC-ROCM. After adjustment for age, diabetes mellitus, and hypertension, the oxygen support was not associated with PC-ROCM (AOR = 1·0; 95% CI = 0·7 to 1·5). Among the cases who received oxygen support, nearly one-third (32%; n = 57) received it through cylinders. Cases had three times higher odds of receiving oxygen support through cylinders (AOR = 3·5, 95% CI = 1·9 to 6·1) than controls. Cases had around five times higher odds of practicing frequent nasal washing than controls (AOR = 4·8, 95% CI = 1·4 to 17·0) after adjusting for age. Presence of end organ damage and type of treatment for diabetes mellitus was not associated with PC-ROCM (S1 Table).

### Among cases and controls never-hospitalized for COVID-19

#### Profile of cases and controls (Table 1)

We included 116 cases and 231 controls. The proportion aged 45–59 years and ≥60 years was significantly higher among the cases (46% and 22%) as compared to the controls (19% and 8%). The majority of the cases than the controls were men (66% vs. 58%), had completed secondary and graduate-level education (66% vs. 83%), and lived in a pucca house (85% vs. 97%). More cases were village residents (43%) than the controls (22%).

#### Analysis of risk factors for PC-ROCM (Table 3)

**Table 3:** Risk factors for mucormycosis among never-hospitalised COVID-19 patients, multi-centric case-control study of post-COVID rhino-orbito-cerebral mucormycosis, India, 2021.

Characteristics		Cases (N = 116)	Controls (N = 231)	Odds Ratio (95% CI)			
				Unadjusted estimate	p value	Adjusted estimate	p value
		n (%)	n (%)				
**SOCIO-DEMOGRAPHIC**							
Age group (years)*	18–44	37 (31·9)	169 (73·2)	Reference			
	45–59	53 (45·7)	44 (19·0)	5·5 (3·2 to 9·4)	<0·001	-	
	≥ 60	26 (22·4)	18 (7·8)	6·6 (3·3 to 13·3)	0·001	-	
**Occupation** ^†^ ^$^	Agricultural	22 (19·0)	22 (9·6)	2·2 (1·2 to 4·2)	0·015	1·8 (0·9 to 3·7)	0·093
	Non-agricultural occupations & unemployed	94 (81·0)	208 (90·4)	Reference			
**CO-MORBIDITY**							
Presence of Diabetes Mellitus^†^		74 (63·8)	36 (15·6)	9·5 (5·7 to 16·0)	<0·001	6·7 (3·8 to 11·6)	<0·001
Presence of Hypertension^‡^		34 (29·3)	26 (11·3)	3·3 (1·8 to 5·8)	<0·001	1·2 (0·6 to 2·4)	0·623
Presence of any other comorbidities^¥^		10 (8·6)	14 (6·1)	1·4 (0·6 to 3·4)	0·384		
**CLINICAL STATUS/MANAGEMENT**							
Elevated plasma glucose level^§^ ^@^		44 (89·8)	15 (62·5)	5·3 (1·5 to 18·3)	0·009	13·7 (2·2 to 84·0)	0·005
Median (IQR) Serum ferritin in μg/L		630^‡^ (400–1000)	172^§^ (40·9–793)		0·384		
**Steroid use** *							
Ever used steroids		74 (63·8)	35 (15·2)	9·8 (5·8 to 16·6)	<0·001	-	
More than 480 mg prednisolone equivalent *{Median dose among controls}*		57 (49·1)	16 (6·9)	16·6 (8·7 to 31·7)	<0·001	-	
Less than 480 mg prednisolone equivalent *{Median dose among controls}*		14 (12·1)	16 (6·9)	4·1 (1·9 to 9·0)	<0·001	-	
Unknown dose		3 (2·6)	3 (1·3)	4·7 (0·9 to 23·9)	0·065	-	
No steroid use		42 (36·2)	196 (84·9)	Reference			
**Antibiotic use** ^†^		94 (81·0)	188 (81·4)	1·0 (0·6 to 1·7)	0·937	-	
More than 9 days of hospitalization *{Median among controls}*		141 (52·8)	120 (46·9)	1·3 (0·9 to 1·8)	0·175	0·8 (0·5 to 1·2)	0·241
**Used other medications**							
Remdesivir		0	0	-	-		
Ivermectin		57 (49·1)	92 (39·8)	1·5 (0·9 to 2·3)	0·108	-	
Hydroxychloroquine		1 (0·9)	6 (2·6)	0·3 (0·04 to 2·7)	0·431	-	
Tocilizumab		0	0	-	-		
Iron supplements		3 (2·6)	3 (1·3)	2·0 (0·4 to 10·2)	0·406	-	
Zinc containing vitamin supplements*		71 (61·2)	160 (69·3)	0·7 (0·4 to 1·1)	0·134	-	
Indian traditional medicines (Ayush)		2 (1·7)	7 (3·0)	0·6 (0·1 to 2·7)	0·723	-	
**Mask use prior to onset of COVID-19**							
Cloth masks only		50 (43·1)	61 (26·4)	2·6 (1·5 to 4·5)	<0·001	-	
Surgical masks only		34 (29·3)	109 (47·2)	Reference			
Both Cloth and Surgical mask		24 (20·7)	61 (26·4)	1·3 (0·7 to 2·3)	0·455	-	
No masks		8 (6·9)	0	Omitted			
**Oxygen use at home through oxygen concentrator/humidifier** ^¶^		7 (6·0)	13 (5·6)	1·1 (0·4 to 2·8)	0·878	0·3 (0·1 to 0·8)	0·017
**Practiced nasal washing** ^#^		7 (6·0)	6 (2·6)	2·4 (0·8 to 7·3)	0·122	2·8 (0·8 to 10·1)	0·116
**Practiced steam inhalation** *		52 (44·8)	139 (60·2)	0·5 (0·3 to 0·8)	0·007		

* No confounders identified;

^†^ Adjusted for age;

^$^1 missing data in controls;

^‡^ Adjusted for age and diabetes mellitus;

^@^ data missing for 67 cases and 207 controls;

^§^ Adjusted for steroid use;

^¶^ Adjusted for age, diabetes mellitus, and hypertension;

^#^ Adjusted for age, and steam inhalation,

^¥^ co-morbidities includes heart disease, chronic kidney disease, cancer, organ transplant, HIV/AIDS, chronic sinusitis

Cases were more likely to be ≥45 years of age. Cases were seven times more likely to have had diabetes mellitus (AOR = 6·7; 95% CI = 3·8 to 11·6) than controls after adjusting for age. After adjusting for steroid usage, those with elevated plasma glucose levels were 14 times more likely to have RC-ROCM (AOR = 13·7; 95% CI = 2·2 to 84·0). Steroid usage was associated with PC-ROCM (AOR for above median levels = 16·6; below median levels = 4·1).

Cases had lower odds of using oxygen concentrators/humidifiers than the controls (AOR = 0·3; 95% CI = 0·1 to 0·8) after adjusting for age, diabetes mellitus, and hypertension. The odds of use of steam inhalation was also lower among the cases than in the controls (OR = 0·5; 95% CI = 0·3 to 0·8). Nasal washing, other medications such as remdesivir, ivermectin, iron/zinc supplementation, and medications from the traditional medical system were not associated with PC- ROCM.

## Discussion

The results of this pan-India case-control study indicates that older age, hyperglycemia, diabetes mellitus, and steroid usage were associated with PC- ROCM, irrespective of hospitalization for COVID-19. Frequent nasal wash and use of oxygen from cylinders were additional risk factors among those ever-hospitalised. In the never-hospitalised group, individuals reporting the use of oxygen through concentrators/humidifiers and practice of steam inhalation were at a lower risk for PC-ROCM.

Hyperglycemia as an independent risk factor for PC-ROCM, irrespective of hospitalization for COVID-19, merits attention. Published reports indicate that COVID-19 is associated with hyperglycemia in general. Hyperglycemia or new-onset diabetes mellitus has been observed with COVID-19 [22] and is hypothesized to predispose to ROCM through phagocyte dysfunction, defective chemotaxis, and impaired intracellular killing of Mucorales [11, 23]. Hyperglycaemia during COVID-19 could be due to SARS-CoV-2 infection *per se* [11] and steroid usage during treatment of COVID-19 [23, 24]. Therefore, it is critical to distinguish the influence of prior diabetic status and hyperglycemia induced by steroid/COVID-19 on PC-ROCM. This emphasises tight monitoring of plasma glucose even among non-diabetic COVID-19 patients.

Diabetes mellitus as a risk factor for ROCM is well documented during the pre-COVID era [5, 25]. In COVID-19 pandemic, pre-existing diabetes could worsen glycaemic control, thereby causing ketoacidosis and increasing the risk of mucormycosis [26, 27]. Our finding is consistent with published studies reporting a higher risk of mucormycosis among people with diabetes and more so after COVID-19 [4, 9, 11, 24, 26–28].

In this study steroid usage was associated with PC-ROCM independent of hospitalisation, irrespective of cumulative dose. We documented substantial steroid usage among all COVID-19 patients and more so among the never-hospitalised group indicating irrational and unmonitored use. Poor adherence to the guidelines on steroid use for COVID-19 has been reported widely in India [29–32]. Hyperglycemia is a known side effect of steroid intake [33, 34]. Before the emergence of COVID-19, steroid usage was not seen as an independent risk factor for mucormycosis [5]. However, studies on COVID-19 associated mucormycosis have reported steroids as an important predisposing factor [4, 8, 22, 24, 25, 28, 35]. The occurrence of mucormycosis among steroid users is mediated through macrophages/neutrophil dysfunction or hyperglycemia [5]. The viral-induced lymphopenia and endothelitis add to the favorable environment produced by steroids, diabetes mellitus, and hyperglycemia in causing COVID-19 associated ROCM. Therefore, COVID-19 patients on steroid treatment need to be monitored for their glycaemic status and educated to recognize and report symptoms and signs of ROCM. Further, prescribing steroids for COVID-19 patients in home isolation or non-hospital care centres needs to be done rationally, along with stringent monitoring and control of plasma glucose levels.

Similar to the widely reported use of steroids, antibiotic overuse and consequent secondary bacterial infections were documented [30, 31]. Antibiotics could potentially eliminate the normal nasal flora and favour fungal growth. However, there was no established association in the present study, where antibiotic usage was high.

In India, people use nasal irrigation with warm saline water at home (either before/after hospitalisation) for symptom relief based on the traditional Indian systems of medicine. Among the ever-hospitalised group, frequent nasal wash (many times a day) was associated with PC-ROCM despite being reported by a small subset. It is possible that repeated nasal washing depletes the commensal organisms of the nasal mucosa and thus allowing the fungus to thrive uninterrupted [32]. Poor maintenance of cloth masks, especially in the humid environment of India, could add to fungus friendly environment, although not specifically explored in this study.

Oxygen requirement during COVID-19 reflects the severity of pneumonia caused by SARS-CoV-2. During the pandemic’s peak, receipt of oxygen and its type depended entirely on the availability of hospital beds. Acute shortage of hospital beds with oxygen support could have been the reason for higher case fatality among severe COVID-19. Hence, it is likely that our participants could have had mild to moderate COVID-19, and such survival bias might have obscured any reported association with oxygen requirement. Among those who received oxygen, we documented a higher likelihood of PC-ROCM among those receiving through oxygen cylinder, albeit based on small sample size. Oxygen cylinders were used when the health care system was overwhelmed over and above that which could be met by piped oxygen supply. We hypothesize that a breach in the infection control practices during these periods could have contributed to the higher risk for PC-ROCM. During the same period, increased use of industrial oxygen for medical purposes was also implicated in the rise of mucormycosis among hospitalised patients [35]. This is contrary to the fact that a high concentration of oxygen does not sustain the growth of fungi [36].

The practise of steam inhalation and the use of oxygen concentrators/humidifiers seemed to reduce the likelihood of PC-ROCM among those isolated at home or in non-hospital settings in our study. A large proportion of study participants reported the practice of steam inhalation. The Indian Ministry of AYUSH (exclusive to India’s traditional medicine) recommended steam inhalation once a day for symptomatic relief during COVID-19 [37]. We did not document any benefit of steam inhalation among the hospitalised participants [25]. Nevertheless, steam inhalation and use of oxygen in non-hospitalised settings could reflect better adherence to COVID-19 protocols and home management. The participants had mild COVID-19, and thus they were not hospitalised. Further, recall of such practices is likely to be better among the non-hospitalised than those hospitalised, attributable to their health-seeking behaviour. Finally, we cannot rule out bias in the effect estimates due to unmeasured or unknown confounders and residual confounding.

### Strengths of our study

We did a multicentric study with representation from all regions of India to strengthen the generalisability of our findings. Internal validity of the study was ensured by systematic training and monitoring of the site investigators and assessment of risk separately in ever- and never-hospitalized groups. We used an unmatched design since matching may complicate the enrolment of the study participants. Instead, we identified confounders (through DAG) and estimated adjusted measures of association. We analysed the role of an exhaustive set of factors linked to PC-ROCM, such as plasma glucose, steroid dose, and behavioural factors, that were hitherto not commonly examined. Statistical analysis of risk factors was based on an a-priori causal framework guided by directed acyclic graphs for the relationship between PC-ROCM and other variables. Thus each of the associations was adjusted separately for the relevant confounders only, which prevented over adjustment by covariates that are not confounders that would have resulted in potentially biased effect estimates due to induced confounding.

### Limitations of our study

Our study had a few limitations. Firstly, the selection of cases could suffer from survival bias as those who survived ROCM were more likely to be recruited. This could bias our estimates in either direction. To increase representativeness and reduce selection bias, the study included individuals from both private and public health facilities, as well as two groups of people participants (never and ever hospitalized), to also include the persons who did not seek medical care for COVID-19. Secondly, although recruited from within the same hospital, many of the cases and controls in the ever-hospitalised group had received COVID-19 treatment elsewhere before their hospitalization for ROCM management. Hence, we could have missed certain risk factors from earlier hospitalization. However, we included only those individuals wherever complete information was available. In addition, misclassification could have occurred for certain factors such as plasma glycaemic status and steroid usage among the ever-hospitalised group. However, that is likely to be non-differential, leading to the association being biased towards the null. Despite this, we could calculate the odds ratios with reasonable precision. Thirdly, among the never-hospitalised group, recall bias and unverifiable level of practices leading to misclassification of key risk factors could have resulted in biased estimates in either direction. Fourthly, we could not directly measure the association of PC-ROCM with the severity of COVID-19 on account of a lack of uniformity in severity grading and irregular documentation across the multiple hospitals where cases and controls were managed. Finally, we could not incorporate factors like HbA1C and BMI, due to substantial missing values.

## Conclusions and recommendations

We concluded that in COVID-19, hyperglycaemia irrespective of pre-existing diabetes mellitus and high steroid usage, was associated with the occurrence of PC-ROCM regardless of hospitalisation for COVID-19. To reduce the risk of PC-ROCM, we recommended adherence to protocols for rational use of steroids and systematic monitoring of plasma glucose levels notwithstanding the diabetic status.

## Supporting information

S1 Table Clinical status and management among those with diabetes mellitus (n, %), multi-centric case-control study of post COVID ROCM, India, 2021. (DOCX)
