# Correction: Hyperglycemia and steroid use increase the risk of rhino-orbito-cerebral mucormycosis regardless of COVID-19 hospitalization: Case-control study, India

**DOI:** 10.1371/journal.pone.0322153

**Published:** 2025-04-04

**Authors:** Manickam Ponnaiah, Sivaraman Ganesan, Tarun Bhatnagar, Mahalakshmy Thulasingam, Marie Gilbert Majella, Mathan Karuppiah, S. A. Rizwan, Arun Alexander, Sonali Sarkar, Sitanshu Sekhar Kar, Tamilarasu Kadhiravan, Aparna Bhatnagar, Prasanna Kumar S., Vivekanandan M. Pillai, Pradeep Pankajakshan Nair, Rahul Dhodapkar, Pampa Ch Toi, Rakesh Singh, Nirupama Kasthuri, Girish C. P. Kumar, Saranya Jaisankar, Vaibhav Saini, Ankita Kankaria, Anuradha Raj, Amit Goyal, Vidhu Sharma, Satyendra Khichar, Kapil Soni, Mahendra Kumar Garg, Kalaiselvi Selvaraj, ShriKrishna B. H., Kranti Bhavana, Bhartendu Bharti, C. M. Singh, Neha Chaudhary, Vijayaravindh R., Gopinath K., Karthikeyan Palaninathan, Simmi Dube, Rita Singh Saxena, Nikhil Gupta, A. Rathinavel, S. Priya, Arulsundareshkumar, Shama A. Bellad, Avinash Kavi, Anilkumar S. Harugop, Kailesh Pujary, Kirthinath Ballala, Sneha Deepak Mallya, Hanumanth M. Prasad, D. Ravi, N. K. Balaji, Raghuraj Hegde, Neha Mishra, Shalina Ray, S. Karthikeyan, Sudha Ramalingam, A. Murali, Sudhakar Vaidya, Mohit Samadhiya, Dhaval Bhojani, Somu Lakshmanan, Sudagar R. B. Singh, Nataraj Pillai, P. Deepthi, K. Banumathi, V. Sumathi, D. Ramesh, Sonam Poonam Nissar, Khushnood M. Sheikh, Manisha N. Patel, Vipul Shristava, Suresh S. Kumar, K. Shantaraman, Rajkamal D. Pandian, Manoj Murhekar, Rakesh Aggarwal, I-MUCOR Study Group Investigators

I-MUCOR study group investigators is not included in the author byline. I-MUCOR study group investigators should be listed as the seventy-eighth author. The contributions of this author are as follows: Conceptualized research goals and aims, Scrubbed and maintained research data, Conducted a research and investigation process, Developed/designed methodology, Involved management and coordination responsibility for the research activity planning and execution, Contributed reagents/materials/analysis tools, Supervised oversight and leadership responsibility for the research, Validated the overall replication/reproducibility of results/experiments and other research outputs, Wrote/ reviewed & edited the paper.

In the Conclusions subsection of the Abstract, there is an error in the second sentence of the first paragraph. The correct sentence is: Rational steroid usage and glucose monitoring may reduce the risk of post-COVID ROCM.
