# Supplementary material for: Hyperglycemia and steroid use increase the risk of rhino-orbito-cerebral mucormycosis regardless of COVID-19 hospitalization: Case-control study, India
Source: PLoS One. 2022 Aug 8;17(8):e0272042. doi: 10.1371/journal.pone.0272042 (PMC9359565; doi:10.1371/journal.pone.0272042)
Supplement: S1 Table — (DOCX) [file pone.0272042.s001.docx]

**Supplement table 1: Clinical Status and management among those with diabetes mellitus (n, %), multi-centric Case-control study of Post COVID ROCM, India, 2021**

| **Characteristics** | **Hospitalized for COVID-19^*^** | | | **Never hospitalized for COVID-19^*^** | | |
| --- | --- | --- | --- | --- | --- | --- |
|  | **Cases**  **(n= 189)** | **Controls (n=84)** | **p value** | **Cases**  **(n= 74)** | **Controls**  **(n= 36)** | **p value** |
| Median Duration of Diabetes Mellitus in months (IQR) | 36 (3, 97) | 73 (27, 128) | <0.001 | 36 (2, 122) | 71 (36, 122) | <0.001 |
| Presence of end organ damage | 23 (12.2) | 13 (15.5) | 0.456 | 7 (9.5) | 2 (5.6) | 0.483 |
| Type of treatment for diabetes Mellitus | | | | | | |
| Insulin | 59 (31.2) | 22 (26.1) | 0.799 | 13 (17.6) | 6 (16.7) | 0.740 |
| Only Oral Hypoglycaemic drugs | 122 (64.6) | 56 (66.7) | 0.604 | 56 (75.7) | 28 (77.8) | 0.760 |
| AYUSH medications | 1 (0.5) | 4 (4.8) | 0.066 | 2 (2.7) | 0 (0) | 0.476 |
| No treatment | 7 (3.7) | 2 (2.4) | Ref | 3 (4.0) | 2 (5.5) | Ref |

^*^The number within the cells indicate frequency with (column %) unless indicated otherwise
